# Music in the loop: a systematic review of current neurofeedback methodologies using music

**DOI:** 10.3389/fnins.2025.1515377

**Published:** 2025-02-28

**Authors:** Alexandre Sayal, Bruno Direito, Teresa Sousa, Neomi Singer, Miguel Castelo-Branco

**Affiliations:** ^1^Coimbra Institute for Biomedical Imaging and Translational Research (CIBIT), University of Coimbra, Coimbra, Portugal; ^2^Siemens Healthineers, Lisbon, Portugal; ^3^Faculty of Sciences and Technology, University of Coimbra, Coimbra, Portugal; ^4^Intelligent Systems Associate Laboratory (LASI), Guimarães, Portugal; ^5^Center for Informatics and Systems of the University of Coimbra (CISUC), University of Coimbra, Coimbra, Portugal; ^6^Faculty of Medicine, University of Coimbra, Coimbra, Portugal; ^7^Sagol Brain Institute and the Department of Neurology, Tel Aviv Sourasky Medical Center, Tel Aviv, Israel; ^8^Institute for Nuclear Sciences Applied to Health (ICNAS), University of Coimbra, Coimbra, Portugal

**Keywords:** neurofeedback, music, interface, reward, BCI

## Abstract

Music, a universal element in human societies, possesses a profound ability to evoke emotions and influence mood. This systematic review explores the utilization of music to allow self-control of brain activity and its implications in clinical neuroscience. Focusing on music-based neurofeedback studies, it explores methodological aspects and findings to propose future directions. Three key questions are addressed: the rationale behind using music as a stimulus, its integration into the feedback loop, and the outcomes of such interventions. While studies emphasize the emotional link between music and brain activity, mechanistic explanations are lacking. Additionally, there is no consensus on the imaging or behavioral measures of neurofeedback success. The review suggests considering whole-brain neural correlates of music stimuli and their interaction with target brain networks and reward mechanisms when designing music-neurofeedback studies. Ultimately, this review aims to serve as a valuable resource for researchers, facilitating a deeper understanding of music's role in neurofeedback and guiding future investigations.

## 1 Introduction

Neurofeedback (NF) is an advanced technique that facilitates the learning of self-regulation of brain activity, with promising potential for therapeutic applications (Sitaram et al., 2017; Direito et al., 2021a; Young et al., 2021). In each session, the NF system acts as a brain-computer interface (BCI) where real-time feedback on one's neuronal function is provided, often in the form of a visual or an auditory interface, and interpreted by the user. Throughout the training, the participant's goal is to learn to voluntarily control the targeted brain function, usually by explicitly applying certain mental strategies (Thibault et al., 2016). The goal of this procedure is to lead to positive functional changes within brain processes or networks underlying motor, cognitive, emotional, or other processes.

**Figure 1:**
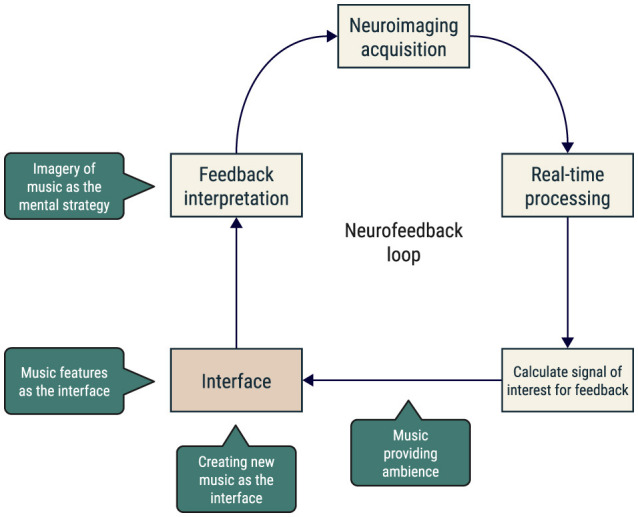
Schematic representation of the neurofeedback loop. The participant's brain activity is recorded, processed, translated via an interface, and fed back to the participant, who interprets this information and acts accordingly. Music could be included in one or more steps of this loop.

Several neuroimaging techniques can be used in an NF loop, namely electroencephalography (EEG) and functional magnetic resonance imaging (fMRI). The analysis of brain activation in real-time was made possible by recent advances in fMRI acquisition and processing software (Christopher deCharms, 2008; Sorger and Goebel, 2020; Koush et al., 2017). In the case of real-time fMRI neurofeedback, one can define one or more brain regions of interest and calculate a measure of activation or connectivity to give back to the participant. The NF loop is closed when the participant interprets this information and acts accordingly, adjusting or persisting on their current neuromodulation mental strategy (Figure 1).

The search for the best, or more efficient, methodology concerning this technique is thriving, with researchers aiming to make it usable in real-world scenarios, including therapeutic settings. One of the factors that preclude the wide use of NF interventions is the relatively high ratio of non-responders—NF success rates vary considerably across studies, and some participants seem to never achieve modulation of their own brain activity (Kadosh and Staunton, 2019).

The task of transmitting the neuronal information as feedback to the participant efficiently is not trivial. The type of feedback information being transmitted is highly relevant (Ivanov and Chau, 2024; Pereira et al., 2023), as is the interface itself, i.e., how the feedback is being presented. Many interfaces have been explored, with visual interfaces being the most widely used (Krause et al., 2017). The classic thermometer, which changes in temperature depending on the activity in a specific region, is widely used mainly due to its simplicity of interpretation (Travassos et al., 2020; Pereira et al., 2019, 2023). Depending on the neurofeedback objective, interfaces that provide emotional content have also been validated: by conveying facial expressions of an avatar as in Direito et al. (2019), by auditory vocalizations with positive or negative associations (Direito et al., 2021b), or as in Cohen et al. (2016) where a complex multi-modal 3D scenario was created and feedback was translated by the unrest level of a virtual waiting room (in which virtual characters become more or less impatient). As was recently demonstrated (Kadosh and Staunton, 2019), psychological variables such as motivation or mood correlate with the success of an NF intervention. As such, the immersiveness of the NF interface is critical. It was postulated that for neuromodulation to be effective in clinical settings, it is important to set a unifying framework that also addresses the interface—the process relevance of the feedback may contribute to the success of regulation and the outcomes of the procedure as a whole (Lubianiker et al., 2019).

Music is a universal, emotion-provoking stimulus. Listening to music involves tracking sound events over time, and consistently engages a multitude of brain systems, including those related to hearing (Koelsch et al., 2018), movement (Levitin et al., 2018), memory (Schulze and Koelsch, 2012), language (Peretz et al., 2015), and affect (Koelsch, 2018). Concerning the latter, studies uncovered that music-induced emotional experiences are associated with the robust engagement of the limbic and reward systems (Gold et al., 2019; Koelsch, 2020; Mas-Herrero et al., 2021). This is because music can elicit strong emotions among listeners (Scherer, 2004; Schubert, 2013; Fuentes-Sánchez et al., 2021; Sammler et al., 2007), ranging from basic emotions such as happiness and sadness or even more complex ones such as tenderness or grief—it creates an emotional echo in the listener (Davies, 2011). As a strategy to shift from prior emotions or for pursuing different scenarios that elicit new emotions, music can act as a powerful tool for emotion regulation (Cook et al., 2019). Indeed, numerous studies have shown that one of the major reasons for individuals to listen to music is for emotion and arousal regulation (Lonsdale and North, 2011; Randall and Rickard, 2017). In addition, there is considerable evidence for a link between music listening/training and brain plasticity, which further supports the potential interest in using it for neurofeedback (Vik et al., 2018; Blum et al., 2017). In Figure 1, we present a schematic representation of the neurofeedback loop, highlighting some examples of how music could be included in the loop.

Previous studies have demonstrated that music activates multiple brain areas, including the auditory cortex, amygdala, and reward-related structures (Koelsch, 2014; Pando-Naude et al., 2021; Zatorre et al., 2007; Gurevitch et al., 2024). Over the years, studies and theoretical frameworks have proposed numerous psychological mechanisms through which music gains its emotional impact, highlighting along different attributes of the music or the musical experience that mediate them (Juslin and Västfjäll, 2008). One prominent theoretical framework suggests that the brain mechanisms underpinning music-related reward are associated with humans' ability to recognize patterns and predict events based on these temporal patterns, as music proficiently exploits our expectations by manipulating melody, rhythm, and more. This predictive process, active during music listening, engages pleasure-related neural networks and triggers the release of dopamine in the reward centers of the brain (Foster Vander Elst et al., 2021; Zatorre, 2024; Salimpoor et al., 2015; Shany et al., 2019). Both the prediction error (predictions about the music itself, e.g. the next chord) and the reward prediction error (the difference between expected and actual outcomes) (Dewiputri et al., 2021) play a role in the rewarding potential of music. Importantly, music represents a highly individual framework, and the musical features that primarily interact with each individual may vary due to cultural and personal context.

Ros et al. (2020) categorized the several mechanisms that drive the effects of NF interventions. Neurofeedback-specific mechanisms are related to training a target neurophysiological variable, while non-specific ones are dependent on the neurofeedback context, but independent of the act of controlling a particular brain signal. General non-specific mechanisms include the common benefits of cognitive training (as well as psychosocial influences, such as placebo responding). There are also repetition-related mechanisms (e.g. test-retest improvement), and natural ones such as spontaneous remission and cognitive development. All of these may interact to generate a greater or lesser overall effect, which has to be understood in the context of control groups and conditions, crucial for assessing the efficacy and specificity of neurofeedback interfaces. These are important to discriminate the effects of each of the above-mentioned mechanisms. Currently, there is no consensus as to which control group and/or condition is best, and the answer depends on what aspects of the neurofeedback-training design one is trying to control for (Sorger et al., 2019; Lubianiker et al., 2019).

**Figure 2:**
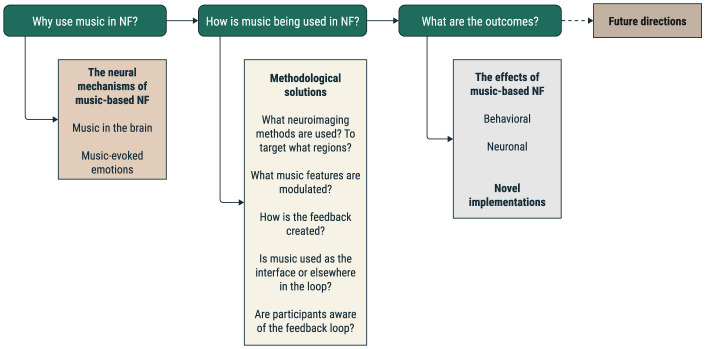
Schematic representation of the review plan. We highlight our three main questions: the motivation for using music in the NF loop, the methodological solutions implemented, and the main findings of the studies.

Here, we review studies that, by acquiring neuronal data in real-time, used music in the context of a neurofeedback intervention, by asking three main questions (Figure 2). The first question focuses on identifying the primary motivations for using music and any specific hypotheses related to music's effects. The second question examines how music is used in the neurofeedback loop, seeking to identify the different paradigms and stages for incorporating music into the loop, such as playing music before, during, or after the task. This analysis could shed light on the optimal ways to use music to elicit specific effects. Finally, the third question looks at the primary outcomes of these studies, aiming to explore the main findings related to the effects of music and its underlying neural correlates. In the end, we discuss possible future directions regarding the use of a musical interface and contribute to the debate regarding music as a valid option in one or more steps of the neurofeedback loop.

## 2 Methods and materials

**Figure 3:**
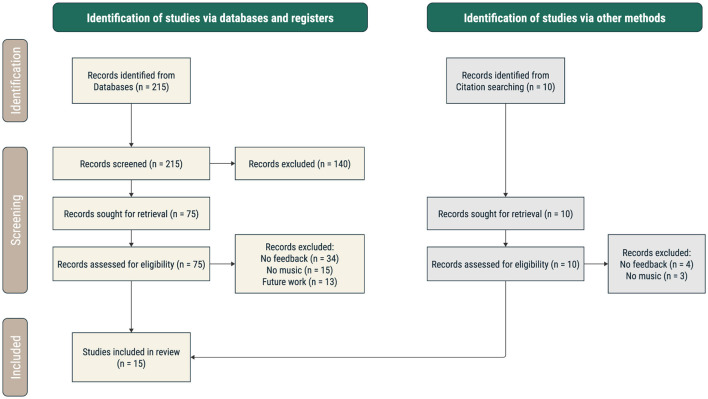
Flowchart of study inclusion according to the PRISMA guidelines.

The literature search presented here follows the guidelines defined in Preferred Reporting Items for Systematic Reviews and Meta-Analysis (PRISMA) (Moher et al., 2009; Page et al., 2021; Haddaway et al., 2022) and was not registered. According to PRISMA, the review protocol includes four stages: identification, screening, eligibility, and inclusion. The search flowchart is displayed in Figure 3 and the PRISMA checklist can be found in Supplementary material.

Statistical analyses and plots were performed using Python. The data was analyzed using descriptive statistics, and the results are presented in the following section.

### 2.1 Identification

The search was performed on Pubmed and bioRxiv with the keywords “music” and “neurofeedback” or “BCI,” finding 103 papers and 112 preprints published from January 2001 up to September 2024. The last search update was made on October 7th, 2024. Additionally, the references of the papers that were selected for full-text eligibility assessment were screened to retrieve further relevant publications.

### 2.2 Screening

The title, objective, neuroimaging technique, and eligibility verdict of the identified articles were recorded on a spreadsheet by AS. Published papers in English were initially filtered by reading the abstract to identify which presented original data, excluding reviews, editorial notes, and conference proceedings, and which implemented a neurofeedback experiment (excluding, for instance, references to neurofeedback in future work).

### 2.3 Eligibility

The following steps were performed by two independent reviewers (AS and BD), each considering half of the records. The decision was based on the input of the two reviewers, while any discrepancies in judgments of risk of bias or justifications for judgments were resolved by discussion to reach a consensus between the two reviewers. A full-text analysis excluded papers that measured only behavioral ratings or that did not include music in the neurofeedback loop. Studies that provided auditory feedback of nonmusical sounds (e.g., sea waves, applause) were also excluded. After this, the references of the selected papers were checked, adding three papers to the list.

### 2.4 Inclusion

The final selection includes 15 studies that fulfill all the requirements (Supplementary Table S1). For these, we extracted the following fields—Title, Authors, Year, Music-specific hypothesis/motivation, Paradigm and music characteristics explored, Changes in NF target region/network and neural correlates of music, Main objective, Neuroimaging technique, Methods summary, Results summary, Main conclusion, Total number of subjects, Music used, Music features, Number of NF sessions, Control groups/conditions, Number of EEG channels/ROIs, Location of music in the loop, Control group type, Music-NF impact, Number of sessions with active feedback, Number of participants in the active group.

## 3 Results

**Figure 4:**
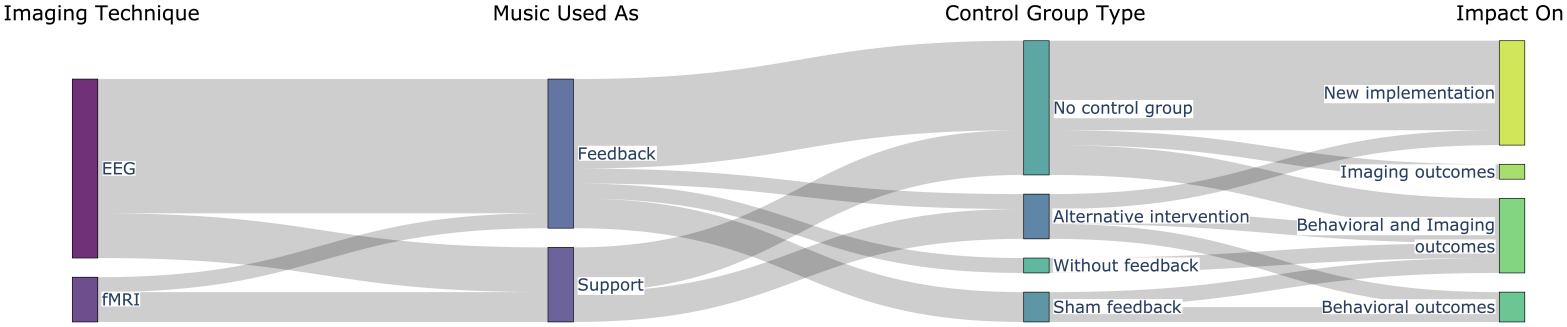
Overview of the distribution of the studies according to the imaging technique, how music was used in the feedback loop, the control groups, and the main outcomes.

The results are organized according to the three research questions: the first question relates to the motivation for using music in the NF loop, the second addresses the methodological solutions implemented until now in music-based NF experiments, and the last summarizes the main findings of the studies considered. The table with the extracted fields from all studies can be found in Supplementary material, while we summarize graphically four of these fields in Figure 4.

### 3.1 Motivation for using music in the NF loop

The main motivation for using music in the context of neurofeedback is explicitly referred to by six of the studies analyzed here: its ability to evoke emotions in the listener can be explored for emotion regulation (Ehrlich et al., 2019; Pino, 2022; Fedotchev, 2018; Ramirez et al., 2015; Pino and La Ragione, 2016; Daly et al., 2016). However, it is unclear if there are specific hypotheses for the mechanism, brain structures, or networks involved that allow music to act on emotion regulation, particularly neurofeedback.

We found clear associations between music and the study objectives. In Takabatake et al. (2021), the objective was to use classical music with different degrees of superimposed white noise to modulate the power of a specific EEG frequency band (in this case, the alpha band as measured in frontal areas) to the point at which some cognitive improvement (namely in a short-term memory test) is achieved. The authors linked the use of music in this NF context with previous evidence from alpha power training methodologies. In a proof-of-concept clinical study (Keller and Garbacenkaite, 2015), the authors mention that for patients with unresponsive awareness syndrome (UWS, a consequence of severe brain injuries), the awareness in the auditory domain was greater than in other domains, inviting the use of auditory stimulation for therapeutic approaches. In this study, three patients listened to favorite songs during a neurofeedback intervention that aimed to determine if they were in some way able to alter their brain activity. In Cordes et al. (2015), the authors recruited patients with schizophrenia and proposed an NF paradigm to enable them to control the activity of their anterior cingulate cortex (ACC), known to be dysfunctional in schizophrenia. Without any previous instruction by the experimenters regarding the mental strategy, the clinical group tended to use the imagery of music as the NF strategy, while the control group tended to use the imagery of sports.

The exploration of music's potential in regulating emotions was also evident in clinical contexts linked to emotion disorders. The pilot study by Ramirez et al. (2015) aimed to allow elderly participants with depression to control two parameters of a set of music performances using their own emotional state, as measured via EEG probes. In Fedotchev (2018), the NF experiment was designed to reduce stress-induced functional disturbances in people who had specialized jobs associated with high workloads.

### 3.2 Methodological solutions

**Figure 5:**
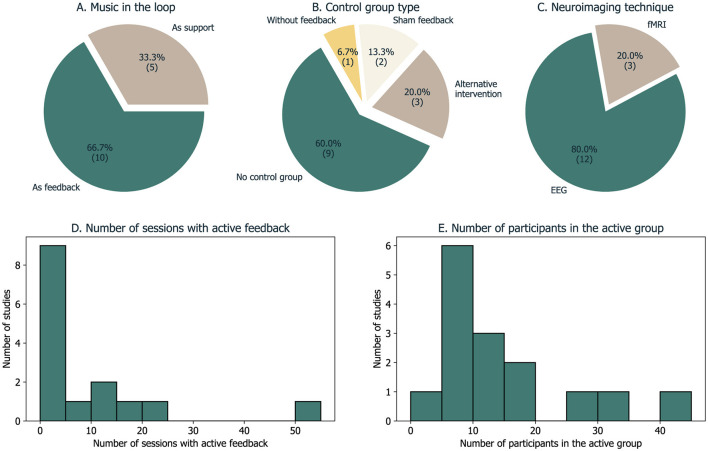
Overview of five methodological fields extracted from the sample of 15 studies. **(A)** How was music used in the feedback loop; **(B)** The type of control group/condition; **(C)** The neuroimaging technique used to record brain signals; **(D)** A histogram of the number of sessions with active/true feedback; **(E)** A histogram of the number of participants that received active/true feedback.

In our systematic review, we identified two distinct categories of methodological solutions that we report in the following sections. In ten cases, music was utilized as feedback directly, whereas, in the other five, another feedback modality was used and music served as a tool to help participants perform during the NF experiment (Figure 5A). At the end, we also address the matter of control groups in these studies.

#### 3.2.1 Music as the interface

Several musical features have been explored for neurofeedback paradigms, such as loudness, tempo, volume, or audio quality. The feedback was conveyed by applying changes in these features based on the neuronal signal. In the following paragraphs, we describe the methodology of the ten studies from our sample that used music directly as a feedback interface.

##### 3.2.1.1 Controlling music features

The pilot study by Ramirez et al. (2015) aimed to allow elderly participants with depression to control the loudness and tempo of music pieces. These were adjusted, in real-time, to the EEG recording from four locations of the prefrontal lobe. The participants were asked to increase the loudness and tempo of the pieces, which were linked to a measure of arousal (beta-to-alpha activity ratio) and valence (right-to-left alpha activity ratio, i.e., frontal alpha asymmetry). The authors relate beta waves to an alertness state and alpha waves to a more relaxed state, hence using their ratio as an indicator of arousal. As for measuring valence, the authors suggested that the left frontal area was more associated with positive memories, while the right frontal area was more associated with negative emotions. They explored this putative interhemispheric difference in the alpha band for estimating valence.

Fedotchev (2018) used an EEG-based neuronal signal to adapt the loudness of the music the participants were listening to. During the EEG session, participants listened to classical music compositions, and the loudness was controlled, in real-time, by the amplitude of the participant's alpha oscillations, a correlate of wakefully relaxed state and internalized attention. The signal was measured in a single electrode on the occipital lobe—the bigger the amplitude, the louder the music. In this study, participants were unaware of this feedback loop—they were simply instructed to listen to the music with their eyes closed. This is known as implicit or non-volitional neurofeedback (Ramot and Martin, 2022).

In Keller and Garbacenkaite (2015), whenever the ratio between the amplitude of theta and beta activity dropped below an automatically set threshold, patients in a State of Unresponsive Wakefulness heard their favorite music (which was defined by their closest family members). A previous study by the same authors demonstrated that this ratio was directly correlated with arousal and attention processes in healthy and brain-injured participants.

Following a lead that indicates the difficulty of concentrating during an active neurofeedback experiment for a long time, the authors in Takabatake et al. (2021) developed a music-based auditory neurofeedback interface that aimed to be intuitive and immersive. The feedback was provided as a continuous overlap of classical music and white noise. Using a headband-type wearable EEG device, the signals from 4 electrodes on the forehead were recorded and preprocessed in real time to extract the power of the alpha band. Every 3 s, the noise level of the auditory feedback was updated according to the normalized alpha band power—the higher the power, the lower the volume of the noise superimposed on the music.

Trost et al. (2024) investigated the feasibility of using live music performances in the neurofeedback system, as it could, according to the authors, maximize the activity in the left amygdala, a key region for music-evoked emotions processing (Koelsch, 2020). The pianists were asked to adjust their live performances, changing pleasantness in real-time, according to the amygdala activity of the listeners. The authors used 12 musical pieces specifically composed for this experiment and then compared the results to the participants' responses to pre-recorded versions of the same pieces.

In the field of sports, Dekker et al. (2014) investigated the ability of alpha power training to enhance the mental abilities of elite gymnasts, focusing on attentional control. Their training setup consists of an EEG-based feedback loop, where the real-time power of the alpha band in the occipital lobe influences the quality of the music that the participants are listening to. Using a high-pass filter, the participants' favorite music sounded thin and distant if alpha levels were low (as the low tones were removed) and rich and full (as usual) if alpha levels were high. This previously validated setup allowed participants to sit comfortably for a few minutes per day while listening to their favorite music (this was considered by the authors the strength of this system). The rationale for considering the occipital alpha band power as a valid target for neurofeedback was based on the hypothesis that it is related to inhibition during selective attention processes. Van Boxtel et al. (2024) expanded on this work by performing this alpha training in groups of football players—the music quality was adapted according to the level of the brain rhythm to train.

##### 3.2.1.2 Creating new music

The next three studies suggest the presentation of new music/notes based on the participant's brain activation patterns. Ehrlich et al. (2019) proposes a non-invasive BCI system that uses music to mediate a person's emotions. The system establishes a closed-loop interaction between the participant's brain responses and the musical stimuli, generating continuous and controllable patterns of synthesized affective music in real time. The automatic music generation algorithm controls parameters that modulate the music's harmonic mode, tempo, rhythmic roughness, overall pitch, and relative loudness of subsequent notes. The authors suggest the use of a classification pipeline to classify positive/negative arousal and valence based on five EEG frequency bands. The classifier's output then informs the auditory stimuli to be presented as feedback and the corresponding musical features. Deuel et al. (2017) describes a new musical instrument called the Encephalophone that uses biofeedback to allow users to control musical notes in real time using EEG signals. EEG signal power from either the posterior dominant rhythm in the visual cortex or from the mu rhythm in the motor cortex was used to create a power scale which was then translated into the eight notes of a musical scale. The participants hear the note produced and can compare it with the target note. Daly et al. (2016) proposed an affective brain-computer music interface (aBCMI) that aims to modulate users' affective states by identifying their current state and using a case-based reasoning system to determine the best approach to shift them toward a target state. This aBCMI integrates a music composition system that can generate new musical stimuli based on five music features: tempo, mode, pitch range, timbre, and amplitude envelope. Different combinations of these features were associated with nine locations in Russel's arousal-valence plane (the combinations of low, neutral, or high arousal and valence) (Russell, 1980). For instance, the authors link high arousal with a larger pitch spread (range of notes), faster tempo, and harder timbres. A combination of ten EEG frequency bands and physiological signals, such as electrocardiogram and respiratory rate, is provided to a classification pipeline based on support-vector machines that perform real-time estimation of the participant's affective state.

#### 3.2.2 Music somewhere else in the loop

The use of music not as an interface but to aid participants in neurofeedback experiments has been explored in five of the studies.

Lorenzetti et al. (2018) showed evidence of the feasibility of a neurofeedback experiment where participants voluntarily modulated their brain activity, as measured using real-time fMRI, while experiencing complex emotions. Musical excerpts were played during the trials to help participants maximize the intensity of the emotions experienced, while the feedback itself was visual and based on the color of the image being displayed. The authors used mild, gentle music to help participants feel tenderness (positive and affiliative, not romantic emotion experienced toward significant others) and eerie distorted music to help them feel anguish (negative and upsetting emotion).

Cordes et al. (2015) conducted an fMRI-based neurofeedback study where 11 patients diagnosed with schizophrenia (the control group included 11 healthy participants) tried to modulate the activity of their ACC. The NF interface was visual, based on an avatar of a human face, which smiled at the participants according to their activity in the ACC. They were simply instructed to augment the smile intensity using a personalized mental strategy, for which some examples were provided. Whilst the results indicated that the cognitive strategies used by the participants (patients and controls) varied considerably, one of these strategies was what the authors categorized as Music (that included thoughts on favorite music, songs, or playing an instrument). Interestingly, Music was used by eight patients but only four controls, with the combined results indicating that the patients were able to modulate the ACC signal using domains that were less impaired, such as cognitive processing and music imagery. In sum, music was used in this feedback loop as the strategy for achieving the modulation of a target ROI.

Pino (2022) developed a prototype brain-computer interface that reads the participant's neuronal responses to music with single-channel EEG and returns flickering lights, in real-time, that match the brain rhythms observed. The protocol is fully detailed in previous work (Pino and La Ragione, 2016). This continuous closed-loop interaction is not explicitly controlled by the participant (the authors refer to this as a passive BCI), hence the feedback is generated based on spontaneous brain activity given the musical context (also known as implicit or non-volitional feedback). The acquired signal was processed and decomposed into frequency bands, each corresponding to a specific colored light. Hypothesizing for a mechanism of brainwave entrainment, the feedback protocol was applied in a sample of 15 participants with depressive and anxiety disorders split into active and control groups – *n* = 7 and *n* = 8, respectively.

Lastly, Leite et al. (2018) present a case study of a game controlled by a BCI based on Steady-State Visually Evoked Potentials (SSVEP). The study aimed to understand how interface elements influence the system performance and how users interact with the game. One of the elements tested is the presence of background music during the game. However, the music and music genre are not specified.

#### 3.2.3 Control groups and design

Six of the studies in our sample used a control group or condition. Its type depended on whether the objective was to assess the feasibility of using music in the feedback loop or the efficacy of the neurofeedback intervention (Figure 5B).

The participants of the control group of Pino (2022) enrolled in a psychoeducation protocol, providing what the authors call an active control group for the experimental group (which received the neurofeedback intervention). The authors assessed and compared behavioral effects between the groups. All participants were recruited based on clinical symptoms linked to depression and anxiety, and control group participants were matched for age and education level to the experimental group.

Takabatake et al. (2021) implemented a crossover design using random (i.e., non-contingent) feedback—this means that all participants performed active (contingent) and random feedback. In both groups, the cognitive functions of all subjects were evaluated before, between, and after each feedback period. The authors used a crossover design to facilitate within-subject comparison, increase efficiency, and minimize participants' number and variability, but they also acknowledge limitations, especially regarding the uncertainty of a washout period in NF studies. A similar approach was followed by Van Boxtel et al. (2024), which also used a crossover design where half the participants received alpha power training and the other half continued usual practice (treatment as usual), and then switched.

Fedotchev (2018) also implemented a crossover design with two music-based treatment sessions: one with active feedback and one without feedback. Half of the participants started with feedback, and the other half without. The advantage of this design is related to the increased efficiency, as it allows for a direct within-subject comparison. In their discussion, the authors compare the decrease of theta EEG power and the increase of alpha EEG power at the end of both therapeutic procedures, as this finding could indicate wakefully relaxed states and internalized attention. The session with EEG feedback showed a positive shift in indicators of the health and mood of the subjects. However, no statistical comparison between sessions is discussed as this study is exploratory.

Cordes et al. (2015) recruited an age- and gender-matched control group of 11 healthy subjects for a group of patients with schizophrenia. Both groups learned to control the same target region (the ACC) known to be dysfunctional in schizophrenia. Since the study focuses on different modulation strategies (one of which is music imagery), we could not establish clear causality between differences at the group level and music-related components of the NF paradigm. However, the authors emphasize that the clinical group mainly used the imagery of music while the control group imagined sports.

A total of 12 athletes completed the intervention in Dekker et al. (2014). In this study, with a double-blind design, half the participants were assigned to the experimental group (that received alpha power training) and half were assigned to the control group, which received random beta power training. The authors made sure to match the groups by several factors, namely age, gender, profession, and ranking, and also by scores of perceived stress, mood, sleep, and social desirability.

### 3.3 Main findings and outcomes

**Figure 6:**
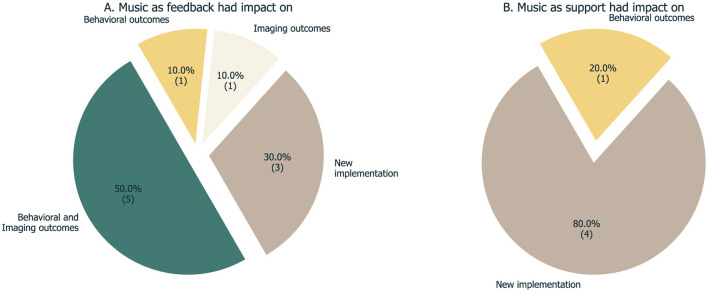
Overview of the outcomes reported by the 15 studies, both for the ones that used music as the interface directly **(A)** and for the ones that used music elsewhere in the loop **(B)**.

The majority of the studies analyzed here report successful results of the intervention or of BCI control achievement. Importantly, we found no reporting of results regarding the use of music as the interface of the neurofeedback—the specificity of the musical interface vs. another type of interface remains untested. We identify success measures in three different domains: modulation of the target brain signal, changes in behavioral/clinical scores, and changes in neural patterns (Figure 6). The number of domains discussed and the level of detail varies among studies. Lastly, we also address the studies that do not associate music feedback with a specific brain signal but rather use music as a complement in the feedback loop.

Five studies report changes in both behavioral/clinical and imaging outcomes. At the end of the intervention of Ramirez et al. (2015), the behavioral depression score applied showed an overall improvement, and the EEG data analysis revealed a decrease in relative alpha activity on the left frontal lobe. While frontal alpha asymmetry is often suggested as a depression biomarker, its clinical diagnostic value is limited, with cross-sectional analyses revealing significant heterogeneity (Van Der Vinne et al., 2017) and very low reproducibility (Kolodziej et al., 2021). Most of these studies were not preregistered, leaving the robustness of this finding yet to be confirmed, particularly considering the analytical flexibility and heterogeneity involved in EEG data analysis. In Fedotchev (2018), the occipital alpha rhythm power increased after the NF intervention session, but significantly so only when the feedback loop was turned on. Both treatment alternatives (with and without feedback) resulted in positive changes in psychological tests. Moreover, post-treatment reports demonstrate acceptance and value in the proposed musical interface, but no additional neural correlates were analyzed. The results presented by Keller and Garbacenkaite (2015) indicate that two out of the three UWS patients were able to drop the theta/beta ratio, measured at the Cz scalp location, during the intervention period, which was mainly a consequence of a decreased theta amplitude (the authors suggest that these results reveal a shift of the dominant rhythm into the alpha band and reflect some brain function recovery). The weekly assessment of the JFK Coma Recovery Scale-Revised (CRSR) increased specifically in the auditory function, motor function, and arousal subscales but, since the sample size was three, the authors only provide a descriptive analysis of the time courses. Nevertheless, this study provides the first evidence that NF can be used in patients with this specific syndrome, but also the potential of music in such an extreme case. In Takabatake et al. (2021), the data analysis from two groups of healthy participants (five subjects per group, receiving real or random feedback) revealed a significant difference in the alpha power, measured at the forehead, achieved during the 4 weeks of intervention when comparing real with random feedback. Cognitive functions were also assessed before and after the intervention (digit span test, standard verbal paired-associate learning test, simple calculation task, N-back test, and Test of Variables of Attention). The results were assessed based on a binary classification of responders and non-responders (based on the regression analysis of a success measure corresponding to the modulation of the target brain signal). The authors report an improvement in short-term memory in responders compared to non-responders. The crossover design and control conditions limit conclusions regarding the causality of the music interface. The training program described in Van Boxtel et al. (2024) was successful in increasing the alpha activity of the participants, as measured by EEG, but also in improving both their performance on task switching and mental rotation tasks and sleep duration, as the results of a self-reported questionnaire revealed.

The authors also report that the participants in the experimental group showed a significant improvement in their performance in a cognitive task compared to the control group. The results suggest that the training program was effective in increasing the alpha power in the occipital lobe of the participants, as measured by EEG. The authors also report that the participants in the experimental group showed a significant improvement in their performance in a cognitive task compared to the control group. The results suggest that the training program was effective in increasing the alpha power in the occipital lobe of the participants, as measured by EEG. The authors also report that the participants in the experimental group showed a significant improvement in their performance in a cognitive task compared to the control group.

In the distinct case of Dekker et al. (2014), the results do not show changes in imaging markers based on the EEG data: a positive change in the occipital alpha power was detected, but it was not significantly different between groups. However, behavioral questionnaires indicated improvements in the level of mental balance, focus ability, and thought control. Although objectively measuring changes in athletes' performance was unfeasible, this study was the first attempt to target the mental capacities of athletes, such as mental shape and focus, with a simple music-based neurofeedback training protocol.

In Ehrlich et al. (2019), changes were found only in the imaging outcomes: the efficacy of the music algorithm was tested on 11 participants in the first study, and the algorithm was embedded in a real-time BCI architecture to investigate affective closed-loop interactions in five participants in a second pilot study. The results suggest that participants were able to intentionally modulate the musical feedback by self-inducing emotions, indicating that the system can capture the listener's current affective state in real-time and potentially provide a tool for listeners to mediate their own emotions by interacting with music. The proposed concept offers a tool to study emotions in the loop, potentially shedding light on emotion-related brain research and clarifying the interactive, spatio-temporal dynamics underlying affective processing in the brain.

Four other studies report the development of a new implementation for music-based NF. The device of Deuel et al. (2017) has been tested on 15 novice users, and the results show that most were able to hit target notes with a level of accuracy significantly higher than random. The Encephalophone is a novel instrument that uses EEG control to create music, and the study suggests that with continued training, users could significantly improve their accuracy and ability to use the instrument. The article discusses the potential benefits of the Encephalophone for both music therapy and neurological rehabilitation. The instrument may be particularly useful for patients who have lost motor function due to conditions such as stroke or traumatic brain injury, as it allows them to generate music using different regions of the brain that are still functioning normally. Overall, the study suggests that the Encephalophone is a promising new technology that could serve as a movement-free musical instrument and as a therapeutic biofeedback device for patients with motor deficits.

Machine learning algorithms were used in two studies to assess imaging data in real time. In Daly et al. (2016), the authors found that the affective music-based BCI can change the majority of participants' affective states to make them happy, calm, or de-stressed. The selected features used by the affective state detection method contain both neuronal and physiological features, indicating the importance of physiological features in identifying affective states. The feasibility of the experiment of Lorenzetti et al. (2018) was proven, with the results showing distinct and relevant brain network fMRI activation for each of the two targeted emotions/trials (tenderness and anguish). As a limitation of the study, the authors present the fact that the same music excerpts were used for all participants. This decision did not account for inter-individual differences in music taste, an important factor that could have affected the intensity of the emotions experienced by each participant. While the behavioral ratings showed that the excerpts helped induce the proposed emotions, the use of personalized audio tracks could have been more effective, as the authors also recognize.

Lastly, Trost et al. (2024) found evidence that live music performances can be used in a neurofeedback system, as the results suggest that the participants' emotional responses to the live music performances were significantly influenced by the performers' ability to adjust the pleasantness of the music in real-time. While the implementation of novel experimental paradigms with live music performances is challenging, the study suggests, based on state-of-the-art connectivity analyzes, that the left amygdala is a key region (a hub) of a dense functional network that is especially sensitive to the emotional content of live music, a feature that could be further explored in future music-based NF interfaces.

Another three studies are based on multimodal approaches and do not link music feedback to brain signals. Pino (2022) combines a repetitive visual and auditory stimulation feedbacking individual's EEG signals as flicker light so that a continuous closed loop can be obtained. The authors showed differences in some sub-scales of the neuropsychological assessment [reduction in depressive symptoms (HAM-D), increase in cognitive function (IQ)] between the two groups, namely when comparing before and after the intervention. Cordes et al. (2015) found that different cognitive strategies were used during neurofeedback targeting the ACC by the two participant groups—patients with schizophrenia reported using the imagery of music. Specifically, imagery of music was used by eight patients and only four controls, while sports was applied by only three patients, but seven controls. However, evidence suggests that the different strategies did not contribute to the differences found in neural activation. Lastly, the authors of Leite et al. (2018) found that the performance of individuals playing their game was not significantly different between the version with background music and the other versions without it. Volunteers reported that background music was almost irrelevant but did not disturb them, suggesting that background sounds do not significantly impact the performance of individuals in this BCI scenario.

## 4 Discussion

In the current paper, we provide a review of the uses of music in the NF context. We highlighted several works in the field and reviewed the main purposes for incorporating music in NF, the various methodological solutions that were implemented in introducing it, the experimental designs that were used to test its efficacy, and the reported outcomes. Overall, while some progress has been made in the research of music-based NF interfaces (Bhavsar et al., 2024), some important issues are limiting their interpretation, specificity, and mechanisms of action. We discuss these in the context of the reviewed studies and hypothesize future directions and developments.

### 4.1 Neural mechanism of music-based NF interfaces

NF interfaces based on music share the conceptual understanding that NF training can support the regulation of functional and structural patterns, promoting neuroplasticity by operant conditioning (Chen et al., 2022; Lubianiker et al., 2019).

The neural correlates of NF training are often divided into two dimensions of NF paradigms, Strategy Execution and Feedback Processing. The Anterior Cingulate Cortex (ACC), the anterior Insula (aI), and the Basal Ganglia (BG) are brain areas associated with the neural mechanisms of NF-assisted self-regulation (Dewiputri et al., 2021). The ACC is functionally connected to the insula and is part of the salience network—a network associated with task-transitioning and monitoring linking cognition and emotion or interoception. The involvement of the BG in NF learning is a well-established concept, and the mechanism involves the dopaminergic pathway. The BG is a target of midbrain dopaminergic neurons, that convey reward prediction error—a neurophysiological signal that relates to how unexpected or surprising an outcome is. This signal is then relayed to cortical structures that evaluate and reinforce or punish behaviors (Skottnik et al., 2019; Paret et al., 2018; Emmert et al., 2016).

The specific mechanisms behind music-based NF interfaces may be related to the emotional impact of music and its capacity to evoke intense pleasure responses. Music-induced pleasantness is directly linked to musical surprises, as it explores reward-related predictive processes, via recruitment of the mesolimbic system (feedback monitoring component) and its connections with the auditory cortex (sensory component) (Shany et al., 2019; Salimpoor et al., 2013). The patterns and pattern variations (surprise) associated with music stimuli elicit prediction errors and reward prediction errors, which trigger pleasure-related neural networks and the release of dopamine in the reward centers of the brain (Koelsch, 2020; Singer et al., 2016; Ferreri et al., 2019; Salimpoor et al., 2011). In Singer et al. (2016), a link was found between the activation of limbic regions, such as the amygdala and the hippocampus, and the affect and variations in temporal information in music. In Trost et al. (2024), the authors found that the amygdala acts as a central node in a dense functional network that is triggered while listening to live music.

Over the years, several mechanisms have been proposed to mediate the link between music and emotion (e.g., Juslin and Västfjäll, 2008), and various musical features and attributes have been highlighted in determining the affective responses to music, such as tempo and loudness' role in arousal, and the musical mode and dissonance level in the level of pleasantness. Our recent study (Sayal et al., 2024) found evidence for a correlation between distinct musical features (notably expressive features such as vibrato and tonal and spectral dissonance) and the brain networks of valence, arousal, and reward. Intriguingly, few of the music-NF studies utilized these known relationships to manipulate the affective tone of the musical pieces introduced in the loop.

Attempts to decode the brain patterns that are revealed when interpreting valence and arousal (Sayal et al., 2024; Daly, 2023; Putkinen et al., 2021) or complex emotions (Koelsch et al., 2021) in music may also provide important insights on the neural mechanisms that could support using music as feedback, guiding design decision in future music-based NF experiments.

Nevertheless, the majority of the studies included in this analysis do not focus on the nature of the feedback and do not evaluate the specificity of the feedback modality. Most studies recognize music's ability to evoke and modulate emotions in the listener, but its specificity and mechanism of action have not been controlled for. The absence of reports on information outside the brain signal of interest limits our ability to evaluate other potentially important patterns such as music-related ones. Measuring whole-brain activation patterns (as Trost et al., 2024 did with a dynamic effective connectivity metric based on fMRI data) will help further reveal the underlying success mechanisms behind music-based NF and control for its specificity by comparing it with other feedback modalities.

### 4.2 Methodological dimensions of music-based neurofeedback

We identified four main dimensions in the implementation of music as the interface in neurofeedback paradigms: (i) the exploration of music attributes, (ii) how they link to the feedback loop, (iii) the imaging modality, and iv. the experimental design and control conditions.

#### 4.2.1 Exploration of music attributes

Music is, by definition, a complex stimulus. In this sense, the characterization of music pieces used in the revised papers varies considerably: we found descriptions based on valence or arousal (Ehrlich et al., 2019; Ramirez et al., 2015), and on true complex emotions such as tenderness and anguish (Lorenzetti et al., 2018). Some studies did not use predetermined music—instead, they asked the participants to select their favorite music to be used as feedback, in an effort to maximize its pleasing/emotional effect (Keller and Garbacenkaite, 2015; Dekker et al., 2014; Van Boxtel et al., 2024). In fact, the use of individually selected music may potentiate the effects of neurofeedback interventions based on music, as recent evidence from brain connectivity suggests (Wu et al., 2019)—familiar and highly rhythmic music generated more and stronger functional connections between the regions of the networks of interest.

The discussion regarding the best solution for music-based interventions is far from settled, as the heterogeneity of approaches reveals.

#### 4.2.2 Linking music to the feedback

The link between musical features and the neurofeedback loop is also a key variable. The studies of Ramirez et al. (2015), Fedotchev (2018), and Dekker et al. (2014) defined features such as loudness and tempo of music pieces selected a priori as feedback properties of interest. These features were adjusted in real-time by coupling them with specific brain signals (e.g. to the power of a certain EEG frequency band or the power ratio between frequency bands). In Keller and Garbacenkaite (2015), a threshold on the theta/beta power ratio was set to start or stop playing specific music pieces tuned to each patient.

An alternative to real-time adjustment of specific features was proposed by Takabatake et al. (2021), as the brain signal of interest was connected to a continuous dynamic overlap of classical music and white noise. A similar approach was used by Van Boxtel et al. (2024), where the effective quality of the music was manipulated by filtering out low frequencies—the lower the level of alpha activity, the more the low frequencies were filtered out, which made the music sound distant and superficial.

Some authors proposed the creation, in real-time, of synthesized affective music (Daly et al., 2016; Ehrlich et al., 2019). This approach allowed for controlling parameters such as the music's harmonic mode, tempo, rhythmic roughness, or overall pitch using neuronal signals. Trost et al. (2024) did this but with live performers—pianists were instructed to adjust the articulation, density of notes and dynamics of the pieces they were playing to make them feel more or less pleasant to the participants. This adjustment was controlled in real-time by the participants' activation of the left amygdala. In the end, they compared the results of using live and recorded piano music as feedback.

Lastly, three studies report the combination of visual and auditory stimuli (Pino, 2022; Leite et al., 2018; Lorenzetti et al., 2018), but the rationale for this implementation is different. In Lorenzetti et al. (2018), the goal was to aid participants maximize the intensity of the complex emotions to be experienced. Leite et al. (2018) added background music to enhance concentration on the task but concluded that it had no impact on the visual task performance. Pino (2022) used auditory stimuli to evoke specific neurophysiological patterns and coupled them with visual feedback.

#### 4.2.3 Imaging modalities

The neurofeedback loop is based on a single neuroimaging modality or combination of two synchronous techniques.

Neurofeedback training is mainly based on EEG data, but with the rapid development of neuroimaging approaches and robust computational tools, fMRI and functional near-infrared spectroscopy (fNIRS) are increasingly being used in closed-loop applications (Thibault et al., 2016). The EEG framework presents several advantages, as it has a quicker setup, is more affordable, directly measures electrical neural activity, and presents a higher temporal resolution. On the opposite, fMRI is a much more expensive, lower temporal resolution modality but with a higher spatial resolution, that also allows measuring BOLD signals in subcortical structures inaccessible to EEG. fNIRS represents a compromise between the two previous techniques as it is cheaper than fMRI, and allows for better spatial resolution than EEG, but is limited to cortical structures (Pinti et al., 2020).

The majority of the studies included in this review are based on EEG data (Figure 5C). The use of EEG was often limited regarding spatial coverages (used a low number of electrodes), which could restrict the analysis of non-specific neural changes associated with the NF task. In turn, fMRI has the potential to provide whole-brain data and a more comprehensive understanding of the mechanisms associated with NF training. However, scalability is important for neurofeedback studies (considering multi-session, longitudinal studies) and the ultimate goal is to promote translation to naturalistic settings. The brain signal of interest in the studies presented here was often based on single channels of interest (often considered frontal channels) related to the purpose considered, particularly to increase focus or to regulate emotional states.

To overcome the limitations of EEG (particularly spatial resolution and access to subcortical structures) one may use computational tools to estimate the brain sources (solving the inverse problem, i.e. determining the signal source spatial position). Recent advances use fMRI-informed EEG models of the activation within a particular region, also known as electrical fingerprint. To develop this EEG-based model of the activation of subcortical structures, the authors use simultaneous EEG/fMRI data to model BOLD signals from these regions using spectro-temporal features from the EEG signal (Simões et al., 2020; Abreu et al., 2020; Singer et al., 2023; Meir-Hasson et al., 2014; Lubianiker et al., 2019).

#### 4.2.4 Music-based NF experimental design and control conditions

Neurofeedback intervention paradigms comprise several parameters, such as the duration of the training/number of sessions and the control groups/conditions, that can have important effects on the training's success and specificity.

The number of sessions with active/true feedback varies considerably in our sample (mean = 9 ± 14), ranging from 1 to 55 sessions (Figure 5D). This illustrates the heterogeneity of criteria regarding the procedure but also that this number depends on the accessibility and cost of the devices and neuroimaging technique. For instance, regarding the imaging technique, the three fMRI studies used one–three sessions, while the EEG studies went up to 55 sessions, five per week.

An appropriate control group is critical to ensure the effectiveness of neurofeedback paradigms. The studies described here vary in the research goals (from more experimental/proof-of-concept studies to clinical efficacy studies) and hence in control groups/conditions. Most studies do not provide any control group—only within-subject changes are studied particularly in the target brain signal (Figure 5B).

There is a lack of consensus over criteria for control conditions in NF literature. The control condition(s) should be determined by the specific research goal of the study and the best procedures that effectively control for relevant confounding factors (Sorger et al., 2019; Lubianiker et al., 2019). Numerous factors should be considered for causality to be unambiguously established (e.g. perception of success, neuropsychological specificity, placebo, and behavioral effects). Moreover, the broad effect of NF training is often related to systemic changes not directly related to the target brain signal (Bassett and Khambhati, 2017).

Proof-of-concept and early phases of development and evaluation of a novel medical intervention may be developed without the implementation of control conditions (usually to explore the action mechanisms involved and not to prove efficacy or specificity). Three studies proposed crossover designs (Takabatake et al., 2021; Fedotchev, 2018; Van Boxtel et al., 2024). In this setting, all participants undergo both active and non-active feedback sessions. Takabatake et al. (2021) implemented a control feedback session with random feedback, while Fedotchev (2018) used a session without feedback. There are important considerations that should be discussed when implementing crossover designs, such as the carryover effect and period effect. These exploratory studies acknowledge the limitations of a crossover design but lack the mathematical formulation to mitigate possible confounds. Dekker et al. (2014) also used sham feedback as a control strategy—the authors translated feedback from another brain signal (beta band power instead of the alpha band power of interest). While some participants should be able to gain a similar level of control over the alternative signal, some might experience frustration effects since no link between the interface and the mental process can be established. Nevertheless, this condition provides control over the contingency between one's own brain modulation and the feedback and therefore controls this important aspect of learning. The success measures in this case were behavioral measures associated with the neural signal of the experimental group, which was key to assessing the efficacy and feasibility of the intervention.

In the case of clinical feasibility studies, Cordes et al. (2015) used two samples—a clinical schizophrenia group and an age- and gender-matched healthy group. Including a control group with healthy participants that undergo the same intervention allowed the exploration of specific mechanisms of action of that intervention in the clinical group, but it is hard to establish the causality of success measures. Pino (2022) recruited a second clinical group to undergo an alternative intervention—this approach allowed them to compare the outcomes of the NF intervention with an already established one but did not address any non-specific effects linked to cognitive training benefits (Sorger et al., 2019).

Regarding the specificity of the music interface and its potential rewarding benefits, none of the studies analyzed here compared different feedback interfaces. In this sense, no conclusions can be drawn regarding the causality between success and the music-based interface.

### 4.3 Results and evaluation of neurofeedback success

The assessment of success in neurofeedback studies usually includes three analysis stages: (i) assess the ability to modulate the target brain signal, (ii) verify the clinical/behavioral effects of the NF training, i.e. the definition and evaluation of outcome measures, and iii. detailed reporting of experimental design variables. The characterization of success is critical to NF literature as many studies have also reported a considerable number of non-responders among participants. A review of 11 studies reports a percentage of non-responders that ranges from 16 to 57%, with a mean percentage of 38% (Alkoby et al., 2018). Considering the sample in our study, we found no consensus regarding the success measure, either in imaging and/or behavioral outcomes (Figure 6). It is important to note that the studies here considered are in different phases of validation, from more exploratory/proof-of-concept (Dekker et al., 2014; Takabatake et al., 2021; Fedotchev, 2018) to more mature studies focusing on clinical validation of a hypothesis (Pino, 2022; Cordes et al., 2015).

First, determining the relationship between the ability to modulate the target brain region and the mechanisms involved in such success is key to a complete comprehension of the framework (Bassett and Khambhati, 2017). For example, network neuroscience provides a flexible and generalizable approach to describe the neurofeedback framework and an explanation of the heterogeneous interaction patterns between its elements. In this sense, a complete characterization of the music stimuli and their neurobehavioral effects is critical to better understand the effect associated with the music-based feedback interface. Study design should consider the neural correlates of music stimuli and their interaction with the target brain signal, neurofeedback, and reward mechanisms to optimize the feedback loop (Singer et al., 2023). Ultimately, this comprehension may lead to the optimization of the framework or contribute to the translation to more naturalistic/clinical setups (e.g. translation from fMRI to EEG using the electrical fingerprint).

Second, verifying the clinical and behavioral effects of NF training requires determining whether these effects are specifically due to NF, i.e., establishing causality between NF learning and changes in clinical or behavioral outcomes. The NF literature has proposed several measures to evaluate the success of NF (Thibault et al., 2018), including assessing the modulation of the target brain signal relative to baseline, the first feedback trial, controls, or as a longitudinal trend in multi-session designs. These imaging-based success measures should, in turn, be associated with improvements in behavioral or clinical outcomes. To strengthen such evaluations, a comprehensive framework should incorporate cognitive, behavioral, and neurophysiological measures. Self-report scales, such as the Profile of Mood States (POMS) and the State-Trait Anxiety Inventory (STAI), could be used to assess changes in emotional and affective states, while cognitive performance might be evaluated using tasks like the n-back task or the Stroop test, which measure working memory and attentional control. Behavioral outcomes, such as stress resilience and relaxation responses, could be captured through validated instruments like the Perceived Stress Scale (PSS) or sleep quality indices. Research must establish whether changes in target brain activity (and its associated networks) causally drive behavioral changes. Appropriate control conditions are critical for determining this relationship. Notably, among the studies reviewed, no statistical evidence has established such causality. This is particularly relevant when considering the role of the music interface, as no control condition has been proposed to isolate its effects. Also, the characterization of the sample regarding music training, for instance, may serve as an important confound for these interventions' success (Zentner and Strauss, 2017). Expanding the range of evaluation indicators would enable a more comprehensive assessment of music neurofeedback's effects and provide a richer reference for future research.

Lastly, to improve the reporting and experimental design standards in the field, the NF research community has proposed the standardization of reporting: the tool “consensus on the reporting and experimental design of clinical and cognitive-behavioral neurofeedback studies” (CRED-NF checklist) (Ros et al., 2020). One item of this checklist addresses preregistration and registered reports, which have previously shown to increase the chance of publishing non-significant findings, effectively decreasing publication bias (Scheel et al., 2021; Allen and Mehler, 2019). Templates and guides for these preregistrations have been proposed for functional MRI (Beyer et al., 2021), EEG (Govaart et al., 2022), and fNIRS (Schroeder et al., 2023) studies.

### 4.4 Future directions

Most NF paradigms analyzed in this study use EEG frequency band power characteristics, particularly alpha, beta, and mu rhythms, and reported good results regarding modulation ability with music-based interfaces. fMRI studies are still scarce, but they allow for a more spatially detailed analysis of the brain regions involved in the NF loop. This could lead to searching network-level metrics that are associated with the success of NF training. In fact, one of the studies reports on the network characteristics associated with the closed-feedback loop (Trost et al., 2024), by analyzing dynamic functional connectivity patterns and concluding that the amygdala may have a hub/central role in the music-evoked emotional experience and feedback. This is key to optimizing music-based NF designs: functional connectivity and brain network definition have developed rapidly in NF research across neuroimaging techniques, with some studies finding evidence for connectivity measures that are related to networks of success monitoring in NF paradigms (Pereira et al., 2023; Trambaiolli et al., 2022).

Neurofeedback training based on EEG is particularly sensitive to transients and noise. Moreover, EEG signals measured by channels are the combination of different sources and the exact neural correlate to the task is often not clear. The solution to determine the signal sources, also known as the inverse problem, requires computationally intensive methods that were not addressed in these studies. Alternatives such as the electrical fingerprint have been proposed in the literature, favoring network-based approaches for NF and highlighting the consideration of individual differences in brain function (Singer et al., 2023; Meir-Hasson et al., 2014; Gurevitch et al., 2024). To improve the signal-to-noise ratio, multi-modal signal acquisition might also be used. Synchronous acquisition of different neuroimaging techniques, such as EEG-fNIRS or EEG-fMRI, allows the combination of the advantages of both techniques— increased time and spatial resolution. Additionally, physiological data can also be combined (e.g. galvanic skin response, electromyography, electrocardiography) not only to regress noise confounds but also as a complementary biofeedback signal [e.g. galvanic skin response is known to be associated to autonomic responses signaling emotional states (Ribeiro et al., 2019; Markiewicz et al., 2022)].

An alternative to volitional neurofeedback, as proposed in the included studies, is the use of non-volitional neurofeedback, also known as covert or implicit neurofeedback. The participant is simply given positive or negative feedback whenever a specific target brain pattern occurs. The idea is to directly reinforce spontaneously emerging brain states of interest (Ramot and Martin, 2022). On the one hand, this approach limits the ability of participants to learn mental strategies to control the activation of specific brain areas. On the other hand, through implicit feedback, we can manipulate spontaneous activity at the network level. The dynamic nature of music feedback, as a naturalistic, highly individual, but also customizable stimuli, could make music an excellent candidate for implicit feedback interfaces.

We found no neurofeedback study exploring the link between music and pain. The effects of music listening on pain perception and reduction are well documented in the literature (Arnold et al., 2024), besides the fact that its clinical relevance is still underexplored (Werner et al., 2023; Sihvonen et al., 2022). The use of music as a neurofeedback interface in future clinical trials targeting pain could be a promising approach to explore the mechanisms of music-induced analgesia and its neural correlates.

## Data Availability

The fields extracted of the studies included in this review are provided in Supplementary material. Further inquiries can be directed to the corresponding author.
