# Supplementary material for: Music in the loop: a systematic review of current neurofeedback methodologies using music
Source: Front Neurosci. 2025 Feb 28;19:1515377. doi: 10.3389/fnins.2025.1515377 (PMC11906423; doi:10.3389/fnins.2025.1515377)
Supplement: Supplementary file 1 [file Table_1.pdf]

ID	Authors	Year	Title	(why) Is there a music-specific hypothesis/motivation?	(how) Paradigm and music characteristics explored	(what) Changes in NF target region/network and neural correlates of music	Study on / Objective	Neuroimaging technique	Methods notes	Main results	Main conclusion	N	Type of article	Music used	Music features	N sessions	Control groups	Number, position of EEG channels / ROIs	Link	Music in the loop	Control group type	Music-NF had impact on	Number of sessions with active feedback	Number of participants in the active group
C005	Pino O.	2022	A randomized controlled trial (RCT) to explore the effect of audio-visual entrainment among psychological disorders	"To assist potential BCI methods for emotion regulation, music is a valuable medium, as it is widely acknowledged to be very effective in eliciting affective responses. Hence, incorporating musical feedback into BCI offers excellent possibility for emotion-regulation systems."	A randomized controlled trial (RCT) to explore the effect of audio-visual entrainment among psychological disorders	--	Explore the efficacy of Neuro-Upper (NU), a BCI prototype aimed at calibration of neural oscillations. NU combines repetitive visual and auditory stimulation feed backing individual's EEG signals as flicker light so that a continuous closed loop can be obtained.	EEG	A BCI prototype was proposed to feedback a person's affective state such that a closed-loop interaction between the participant's brain responses and the musical stimuli is established. It feedbacks in real-time flickering lights matching with the individual's brain rhythms undergo to auditory stimuli	Outcome measures revealed either a significant decrease in Hamilton Rating Scale for Depression (HAM-D) scores and gains in cognitive functions only for participants who undergone to the experimental treatment.	future work needs to validate the relationship proposed here between music and brain responses. Findings of the present study provided support to a range of research examining BCI brain modulation and contributes to the understanding of this technique as instruments to alternative therapies	15	Original	Playlists without restrictions to the genre of music, which included classical, folk, jazz, electronica, rock, punk, techno and tango	--	55 (5 per week)	Active control group (n=8) had face-to-face sessions of psychoeducatio n	1 channel (FP1)	https://doi. org/10. 23750/abm. v92i6.12089	support	alternative	visual	55	8
C012	Takabatake K, Kunii N, Nakatomi H, Shimada S, Yanai K, Takasago M, Saito N.	2021	Musical Auditory Alpha Wave Neurofeedback: Validation and Cognitive Perspectives	The music-based auditory feedback increases te power of the alpha wave	The music-based auditory feedback increases te power of the alpha wave	Alpha power and cognitive functions (behav) were assessed along the 16 days of NF. Differences found.	to clarify whether music-based auditory neurofeedback increases the power of the alpha wave in healthy subjects	EEG	During neurofeedback, white noise was superimposed on classical music, with the noise level inversely correlating with normalized alpha wave power.	A crossover analysis showed that normalized alpha-power was significantly higher in NF than in Random Feedback; therefore, music-based auditory neurofeedback facilitated alpha wave induction.	The present study employed a long period of auditory alpha neurofeedback and achieved successful alpha wave induction and subsequent improvements in cognitive functions.	10	Original	Air on the G String (Bach)	--	16 sessions (1 per day)	Cross-over design. Random feedback	4 channels (2 in the forehead, 2 in dorsal side of auricles)	https://doi. org/10. 1007/s10484-021-09507-1	feedback	sham	both	8	10
C019	Ehrlich SK, Agres KR, Guan C, Cheng G.	2019	A closed-loop, music-based brain-computer interface for emotion mediation	To aid potential BCI approaches for emotion regulation, music isauseful medium, as it iswidely acknowledged to be highly effective in eliciting affective responses. Indeed, one of the main reasons people reportedly listen to music isto change or enhance their mood. Therefore, incorporating musical feedback into BCI systems offers great potential for emotion-regulation systems.	70 band-power features translated into arousal and valence	The results show that music feedback modulations towards the happy state were accompanied by significant power decrease in beta band over frontal areas, in particular in the high beta band (21-30Hz), as well as asignificant increase in gamma band power over the fronto-central right hemisphere.	In the first study, we tested the efficacy of our music algorithm by measuring subjective affective responses from 11 participants. In a second pilot study, the algorithm was embedded in a real-time BCI architecture to investigate affective closed-loop interactions in 5 participants.	EEG	The automatic music generation system was implemented as a rule-based probabilistic algorithm. Our algorithm generates streams of MIDI-events, whereupon the occurrence and type of events are modulated by several continuous control parameters. These control parameters were implemented such that they modulate the musical pattern according to five music structural components, namely: harmonic mode, tempo, rhythmic roughness, overall pitch, and relative loudness of subsequent notes.	Preliminary results suggested that participants were able to intentionally modulate the musical feedback by self-inducing emotions (e.g., by recalling memories), suggesting that the system was able not only to capture the listener's current affective state in real-time, but also potentially provide a tool for listeners to mediate their own emotions by interacting with music.	The proposed concept offers a tool to study emotions in the loop, promising to cast a complementary light on emotion-related brain research, particularly in terms of clarifying the interactive, spatio-temporal dynamics underlying affective processing in the brain.	5	Original	Music can be thought of as acombination of harmonic, rhythmic and timbre components that change over time. The automatic music generation system was implemented as arule-based probabilistic algorithm. Our algorithm generates streams of MIDI-events, whereupon the occurrence and type of events are modulated by several continuous control parameters.	harmonic mode, tempo, rhythmic roughness, overall pitch, and relative loudness	2	--	14 (AF3, AF4, F7, F8, F3, F4, FC5, FC6, T7, T8, P7, P8, O1, O2)	https://doi. org/10. 1371/journal. pone.0213516	feedback	none	imaging	2	5
C024	Lorenzetti V, Melo B, Basílio R, Suo C, Yücel M, Tierra-Criollo CJ, Moll J.	2018	Emotion Regulation Using Virtual Environments and Real-Time fMRI Neurofeedback	--	The color of the virtual environment was used as BCI changed in real time with increased engagement of the neural activity/pattern corresponding to distinct target emotional states—orange for tenderness trials, purple for anguish trials and natural light tones for neutral trials. Participants were instructed to experience tenderness or anguish as intensely as possible in the respective trials and to increase the intensity of their emotion to turn in real time, the color of the virtual environment BCI to as orange as possible during tenderness trials, and as purple as possible during anguish trials.	--	Using rtfMRI-NFB, we enabled participants to voluntarily enhance their own neural activity while they experienced complex emotions	fMRI	Musical excerpts were played during the trials to aid participants maximize the intensity of the emotions experienced. In this case, mild gentle music was played while feeling Tenderness, and eerie distorted music while feeling Anguish.	participants successfully increased in real time, the activity of the septo-hypothalamic area and the amygdala during the ROI based rtfMRI-NFB, and successfully evoked distributed patterns of brain activity classified as tenderness and anguish during SVM-based rtfMRI-NFB	Our multimodal VR/rtfMRI-NFB protocol provides an engaging tool for brain-based interventions to enhance emotional states in healthy subjects and may find applications in clinical conditions associated with anxiety, stress and impaired empathy among others.	8	Original	Music excerpts were fixed for each trial type, and not influenced by current neural/psychological states (no music for Neutral, mild, gentle music for Tenderness and eerie, distorted music for Anguish). Music excerpts were selected from 20 audio tracks, all normalized using the root mean square feature of Audacity software. The audio tracks were previously rated to have comparable volume, pace, and rhythm. For the rtfMRI-NFB task runs, four excerpts for tenderness and four excerpts for anguish were played.	The SVM rtfMRI-NFB method used a feature selection mask that included brain regions implicated in positive affiliative emotions (e.g., frontal, temporal, parietal and subcortical areas), and that excluded from SVM training and decoding those areas involved in sensorimotor or visuospatial processing	2	--	Septohypothala mic area, amygdala, precuneous, DLPFC	https://doi. org/10. 3389/fneur. 2018.00390	support	none	new	2	8

ID	Authors	Year	Title	(why) Is there a music-specific hypothesis/motivation?	(how) Paradigm and music characteristics explored	(what) Changes in NF target region/network and neural correlates of music	Study on / Objective	Neuroimaging technique	Methods notes	Main results	Main conclusion	N	Type of article	Music used	Music features	N sessions	Control groups	Number, position of EEG channels / ROIs	Link	Music in the loop	Control group type	Music-NF had impact on	Number of sessions with active feedback	Number of participants in the active group
C025	Leite HMA, de Carvalho SN, Costa TBDS, Attux R, Hornung HH, Arantes DS.	2018	Analysis of User Interaction with a Brain-Computer Interface Based on Steady-State Visually Evoked Potentials: Case Study of a Game	--	Music used as background only.	--	analyzes a BCI system based on Steady-State Visually Evoked Potentials (BCI-SSVEP) from the perspective of HCI, in such a way as to understand how the elements of the interface affect the user and how the interaction occurs.	EEG	--	Still in relation to sound effects, the amount of collected coins was not significantly different between Version 3, with background music, and the other versions of the game without background music. In the perception questionnaire, the volunteers reported that background music was almost irrelevant but it does not disturb either	This research directs developers to understand users' difficulties and how the interaction of the user with a BCI based on SSVEP occurs	30	Original	--	--	1	--	16 (O1, O2, Oz, POz, Pz, PO4, PO3, PO8, PO7, P2, P1, Cz, C1, C2, CPz, and FCz)	https://doi.org/10.1155/2018/4920132	support	none	visual	1	30
C027	Alexander F.	2018	Stress Coping Via Musical Neurofeedback	A conceptual framework for music-based biofeedback has been presented to show that the use of music, and musical principles, can have a major added value, on top of mere sound signals, to the benefit of psychological and physical optimization of sports and motor rehabilitation tasks.	Revealed alpha EEG oscillator of the individual was used then in 1 of 2 treatment sessions for feedback control of music presentation. Due to specially developed software, music sound intensity was online controlled by current amplitude of alpha EEG oscillator: the bigger its amplitude, the more music loudness, and vice versa	The results demonstrated that in both sessions the power of theta EEG rhythm decreased to the end of the therapeutic procedure, whereas the power of alpha-rhythm increased. However, statistically significant ($P < .01$) changes of alpha EEG power occurred only in the case of feedback EEG control of music loudness. Under both treatment sessions, positive changes in all indicators of SAN test were revealed. Again, the most marked and significant changes occurred only in the case of feedback EEG control of music loudness.	This pilot study aimed to examine the efficiency of music stimulation online controlled by feedback signals from patient's EEG oscillators for the correction of stress-induced functional disturbances.	EEG	loudness of music being controlled by amplitude of alpha EEG oscillator	normalization of the EEG, reduction of stress sensations, and positive shifts in mental and emotional status of the patients were observed. However, statistically significant positive changes occurred only in the case of feedback EEG control of music loudness	After further elaboration, a proposed and tested approach may be used in a wide range of rehabilitation procedures and for stress coping.	16	Original	Popular classical music by Mozart, Bach, Schubert, and others. The finished length of presented audio file was 15 minutes. The same audio file was used in all experiments.	--	2	1 session without feedback	1 (O1)	https://doi.org/	feedback	without	both	1	16
C047	Ramirez R, Palencia-Lefler M, Giraldo S, Vamvakousis Z.	2015	Musical neurofeedback for treating depression in elderly people	"Music activities (both passive and active) can affect older adults' perceptions of their quality of life, valuing highly the non-musical dimensions of being involved in music activities such as physical, psychological, and social aspects (Coffman, 2002; Cohen-Mansfield et al., 2011)."" merely listening to music and speech after neural damage can induce long-term plastic changes in early sensory processing, which, in turn, may facilitate the recovery of higher cognitive functions.""our main goal is to investigate the emotional reinforcement capacity of automatic music neurofeedback systems, and its effects for improving depression in elderly people."	MUSIC FEATURES which allows users to manipulate expressive parameters in music performances using their emotional state. The resulting coordinate is then used to change expressive aspects of music such as tempo, dynamics, and articulation. Prior to the first session, the participants in the study were interviewed in order to determine the music they liked and to identify particular pieces to be included in their feedback sessions- for each participant a set of 5–6 music pieces was collected from commercial audio CDs. PARADIGM Participants were encouraged to increase the loudness and tempo of the pieces so the pieces sounded "happier." As the system was tuned so that increased arousal corresponded to increased loudness, and increased valence corresponded to increased tempo, participants were encouraged to increase their arousal and valence, in other words to direct their emotional state to the high-arousal/positive-valence quadrant in the arousal-valence plane (see Figure 2).	Valence and arousal computation was adapted from Ramirez and Vamvakousis (2012), where the authors show that the computed arousal and valence values indeed contain meaningful user's emotional information. RESULTS EEG data of the participants showed a significant decrease of relative alpha activity in their left frontal lobe ($p = 0.00008$), which may be interpreted as an improvement of their depression condition	introduce a new neurofeedback approach, which allows users to manipulate expressive parameters in music performances using their emotional state	EEG	The neurofeedback system was tuned so that increased arousal, computed as beta to alpha activity ratio in the frontal cortex corresponded to increased loudness, and increased valence, computed as relative frontal alpha activity in the right lobe compared to the left lobe, corresponded to increased tempo.	--	analysis of the collected EEG data of the participants showed a significant decrease of relative alpha activity in their left frontal lobe ($p = 0.00008$), which may be interpreted as an improvement of their depression condition.	10	Original	Each participant select 5/6 musics (see Table 1)	Loudness and tempo	10 sessions (2/week) - 15 minutes each	n/a	14 EEG channels, and a wireless amplifier. The electrodes were located at the positions AF3, F7, F3, FC5, T7, P7, O1, O2, P8, T8, FC6, F4, F8, AF4- FEEDBACK was computed based on: EEG signal was measured in four locations (i. e., electrodes) in the prefrontal cortex: AF3, AF4, F3, and F4 (see Figure 1).	https://doi.org/10.3389/fnins.2015.00354	feedback	none	both	10	10
C048	Cordes JS, Mathiak KA, Dyck M, Alawi EM, Gaber TJ, Zepf FD, Klasen M, Zvyagintsev M, Gur RC, Mathiak K.	2015	Cognitive and neural strategies during control of the anterior cingulate cortex by fMRI neurofeedback in patients with schizophrenia	""we expected differences between the groups with respect to the neural pattern of activation as well as the applied cognitive strategies." (Cordes et al., 2015, p. 2)" "Some template strategies from different cognitive domains were named, i.e., positive autobiographic memories, picturing oneself doing sports or playing an instrument, and concentrating on certain perceptions like feeling the temperature of one's own left foot. However, it was clarified that the subjects needed to find individual ways and strategies to achieve successful regulation of the feedback signal, and that they would be asked to report what kind of strategies they applied after each feedback run as well as in an interview at the end of every training day.	"They were provided with a standardized protocol containing information on the hemodynamic delay and the instruction to only switch in between different strategies after trying one for at least 10 s"	"Cognitive strategies involving music (blue circles) were mostly used by patients and led in some of them but not in controls to high dorsal activation." "MRI NF training of ACC activity in patients with schizophrenia led to activation of the dorsal ACC subsection, whereas controls activated the rostral subsection. In addition, different cognitive strategies were reported, i.e., related to music in patients with schizophrenia and to sports in healthy controls. The difference in strategies, however, did not contribute to the difference in neural activation."	conducted NF training based on real-time functional magnetic resonance imaging (fMRI) in patients with schizophrenia to enable them to control their ACC activity	fMRI	During the regulation blocks, the momentary BOLD activation was fed back to the participants via a BCI providing social rewards. In short, the avatar of a dark-haired male human smiled at the participants with rising intensity when the activity of the ACC increased. In contrast, it gradually returned to a neutral expression when the activity decreased.	Both groups learned to control the activity of their ACC but used different neural strategies: patients activated the dorsal and healthy controls the rostral subdivision. Patients mainly used imagination of music to elicit activity and the control group imagination of sports.	These data emphasize that for therapeutic interventions in patients with schizophrenia compensatory strategies may emerge. Specific cognitive skills or specific dysfunctional networks should be addressed to train impaired skills.	22	Original	Imagery	n/a	three NF sessions	We investigated 11 patients with a confirmed diagnosis of schizophrenia (five females) with a mean age of 38.9 ± 9.3 years, and an age- and gender-matched control group of 11 healthy subjects	a priori defined ACC mask	https://doi.org/10.3389/fnbeh.2015.00169	support	alternative	visual	3	11

ID	Authors	Year	Title	(why) Is there a music-specific hypothesis/motivation?	(how) Paradigm and music characteristics explored	(what) Changes in NF target region/network and neural correlates of music	Study on / Objective	Neuroimaging technique	Methods notes	Main results	Main conclusion	N	Type of article	Music used	Music features	N sessions	Control groups	Number, position of EEG channels / ROIs	Link	Music in the loop	Control group type	Music-NF had impact on	Number of sessions with active feedback	Number of participants in the active group
C049	Keller I, Garbacenkaite R.	2015	Neurofeedback in three patients in the state of unresponsive wakefulness	“Music, on the other hand, is a powerful treatment medium with inherent emotional and non-verbal nature which makes it a perfect stimulus for patients with strongly impaired verbal processing and/or cognition (Noda et al. 2003; Ribeiro et al. 2014).” most awareness in the auditory domain	The quotient of the RMS-amplitude of theta and beta activity was used as a feedback parameter modified the threshold level so that the theta/beta ratio level was below the threshold for 70 % of the time. For the feedback we used samples of patients’ favorite music, recommended by their closest family members (German folk music for patient 1, classical music for patient 2 and a mixture of pop-songs for patient 3). Whenever the theta/beta ratio dropped below the threshold, patients heard their individual music through the loudspeakers positioned at a distance of about 1 meter from the head.	Patient 1’s theta/beta ratio decreased over time, whereas beta amplitudes increased and theta amplitudes decreased. A similar result was obtained for patient 2, the theta/beta ratio and theta amplitude decreased over time. In contrast to patient 1, beta amplitude of the patient 2 remained near the same level during all sessions. Amplitudes of patient 3 highly fluctuated, with a slight decrease of the theta/beta ratio and an increase of the theta and beta amplitudes.	determine whether unresponsive wakefulness syndrome (UWS) patients are able to alter their brain activity using neurofeedback (NFB) technique	EEG	Using an automatic threshold function, patients heard their favourite music whenever their theta/beta ratio dropped below the threshold	Two patients showed a decrease in their theta/beta ratio and theta-amplitudes during this period. The third patient showed no systematic changes in his EEG activity.	the first evidence that NFB can be used in patients in a state of unresponsive wakefulness.	3	Original	For the feedback we used samples of patients’ favorite music, recommended by their closest family members (German folk music for patient 1, classical music for patient 2 and a mixture of pop-songs for patient 3).	ON/OFF (Whenever the theta/beta ratio dropped below the threshold, patients heard their individual music through the loudspeakers positioned at a distance of about 1 meter from the head.)	Each patient received 15 NFB-sessions in 3 weeks.	n/a	“The Cz scalp location served as the” (Keller and Garbacenkaite, 2015, p. 351)	https://doi.org/10.1007/s10484-015-9296-7	feedback	none	both	15	3
C054	Dekker MK, van den Berg BR, Denissen AJ, Sitskoorn MM, van Boxtel GJ.	2014	Feasibility of eyes open alpha power training for mental enhancement in elite gymnasts	“alpha power training by music teaches athletes to (1) learn to self-regulate their brain activity,” The main aim of the present study was to investigate the effects of alpha power training on mental capacities of the athletes, such as attentional control. For this aim, brain activity (by quantitative electroencephalography; qEEG), and mental capacities, such as focus and self-confidence, mood, stress and sleep (by behavioural measures), were measured.	The feedback procedure resulted in a very intuitive feedback mechanism in which a person’s own wellknown favourite music sounded thin and distant if alpha levels were low; and rich and full when alpha levels were high.	There was no main effect of Group (F (1, 12) = 0.00, P = 0.98), nor of Effect Measurement (F(1, 165) = 0.66, P=0.42). As is shown in Figure 1, Group A shows an overall (including all positions) positive change from pre- to post-training measurement, as we expected, although not significantly different from group B (Group × Effect Measurement interaction: F(1, 165) = 0.64, P = 0.43).	we hypothesised that eyes open alpha power training by music teaches athletes to (1) learn to self-regulate their brain activity, and (2) learn to increase their baseline alpha power, herewith improving mental capacities such as focusing the allocation of attention	EEG	The training system employed in the present study uses a direct feedback mechanism on the alpha power in the individual alpha frequency band (IAF ± 2 Hz), which is presented by the quality of music that can be heard through a headset equipped with water-based electrodes	Results indicate small improvements in sleep quality, mental and physical shape	this novel training method can be promising. Because gymnastics is one of the most mentally demanding sports, we value even small benefits for the athlete and consider them indicative for future research.	12	Original	The feedback procedure resulted in a very intuitive feedback mechanism in which a person’s own wellknown favourite music sounded thin and distant if alpha levels were low; and rich and full when alpha levels were high.	quality of music that can be heard through a headset equipped with water-based electrodes	10 training sessions	beta power training instead of alpha power training	The qEEG measurements were acquired by a BioSemi® ActiveTwo amplifier system (Amsterdam, the Netherlands) using a head cap with 32 actively shielded electrodes	https://doi.org/10.1080/02640414.2014.906044	feedback	sham	behavioral	10	6
R001	Deuel TA, Pampin J, Sundstrom J, Darvas F.	2017	The Encephalophone: A Novel Musical Biofeedback Device using Conscious Control of Electroencephalogram (EEG)	--	--	--	A novel musical instrument and biofeedback device was created using electroencephalogram (EEG) posterior dominant rhythm (PDR) or mu rhythm to control a synthesized piano, which we call the Encephalophone.	EEG	Alpha-frequency (8–12 Hz) signal power from PDR in the visual cortex or from mu rhythm in the motor cortex was used to create a power scale which was then converted into a musical scale, which could be manipulated by the individual in real time.	All 15 subjects were able to perform more accurately (average of 27.4 hits, 67.1% accuracy for visual cortex/PDR signaling; average of 20.6 hits, 57.1% accuracy for mu signaling) than a random note generation (19.03% accuracy).	The Encephalophone may have potential applications both as a novel musical instrument without requiring movement, as well as a potential therapeutic biofeedback device for patients suffering from motor deficits	15	Original	include in <Music composition>	it is not a neuro feedback experiment, but during the training of the new device, the participant receives positive/negative feedback in the form of a major chord/tritone, respectively	--	--	--	https://doi.org/10.3389/fnhum.2017.00213	feedback	none	new	1	15
R002	Olimpia Pino, Francesco La Ragione	2016	A Brain Computer Interface for Audio-Visual Entrainment in Emotional Regulation: Preliminary Evidence of its Effects	Music with certain rhythmic parameters is capable of triggering specific brain waves and physiological responses	--	--	The study proposes a BCI in which a headset is connected to a EEG-based neuro-feedback computer so as to administer repetitive audio-visual stimulation or entrainment for regulating the emotional states of individuals with anxiety and depressive disorders.	EEG	--	--	--	7	Original	Playlists without restrictions to the genre of music, which included classical, folk, jazz, electronica, rock, punk, techno and tango	Music to aid feedback, but also as the stimulus	--	--	--	http://www.oiirj.org/oiirj/mar-apr2016/05.pdf	support	none	new	1	7
R003	Daly I,Williams D, Kirke A, Weaver J,Malik A, Hwang F, etal.	2016	Affective brain—computer music interfacing.	A system such as an affective brain–computer music interface (aBCMI) might be useful for such work by facilitating patients who are not musically confident or competent enough to engage in traditional music-making activities as part of the therapeutic process (e.g., performing or improvising new music). Moreover, by enabling the generation of music which matches the emotional state of a patient, an aBCMI might potentially be of use as an expressive tool for patients to express their emotional state to the therapist regardless of physical ability or communicative handicap (for example, patients with autism, Asperger’s syndrome, or even locked-in patients with little or no physical mobility).	--	--	develop and evaluate an affective brain–computer music interface (aBCMI) for modulating the affective states of its users	EEG	An aBCMI is constructed to detect a user’s current affective state and attempt to modulate it in order to achieve specific objectives (for example, making the user calmer or happier) by playing music which is generated according to a specific affective target by an algorithmic music composition system and a casebased reasoning system.	--	Our system represents one of the first demonstrations of an online aBCMI that is able to accurately detect and respond to user’s affective states. Possible applications include use in music therapy and entertainment.	8	Original	--	--	--	--	--	https://doi.org/10.1088/1741-2560/13/4/	feedback	none	new	1	8

ID	Authors	Year	Title	(why) Is there a music-specific hypothesis/motivation?	(how) Paradigm and music characteristics explored	(what) Changes in NF target region/network and neural correlates of music	Study on / Objective	Neuroimaging technique	Methods notes	Main results	Main conclusion	N	Type of article	Music used	Music features	N sessions	Control groups	Number, position of EEG channels / ROIs	Link	Music in the loop	Control group type	Music-NF had impact on	Number of sessions with active feedback	Number of participants in the active group
D004	van Boxtel GJM, Denissen AJJM, de Groot JA, Neleman MS, Vellema J, Hart de Ruijter EM.	2024	Alpha Neurofeedback Training in Elite Soccer Players Trained in Groups	--	The participants listened to their own favorite music that they selected before the start of the study, using earplugs or headphones. The music was passed through a high-pass filter that removed the low frequencies in the music based on the EEG alpha level of the brain signals. The lower the level of alpha activity, the more low frequencies were filtered out. This made the music sound distant and superficial if the alpha level was low, versus and full and rich when the alpha level was high, thus providing an intuitive feedback on the EEG alpha level based on the quality of the music.	The training program resulted in an increase of 34% in alpha activity associated with the training, and improved the athletes' performance on task switching and mental rotation tasks. In addition, self-reported sleep duration, as well as scores on the Being in Shape questionnaire (Feeling of Control and Flow) also improved.	Neurofeedback training is applied in the world of sports as a means to improve athletes' performance. Training sessions are usually organized on an individual basis, one at a time. Here we investigated if the training could also be organized in groups.	EEG	The brain's alpha activity was trained using music-based neurofeedback in a crossover design. A training session consisted of alternating periods of neurofeedback and execution of cognitive tasks.	The training program resulted in an increase of 34% in alpha activity associated with the training, and improved the athletes' performance on task switching and mental rotation tasks. In addition, self-reported sleep duration, as well as scores on the Being in Shape questionnaire (Feeling of Control and Flow) also improved.	This study shows that neurofeedback training is feasible in groups of athletes, which can stimulate its application in team sports.	41	Original	The participants listened to their own favorite music that they selected before the start of the study	music quality	20	cross-over design with treatment as usual	The band is placed horizontally around the head above the ears, so that the electrodes are roughly located above 10–20 positions T3, T4, O1 and O2, all referred to the ground located on the forehead (roughly Fpz).	https://doi.org/10.1007/s10484-024-09654-1	feedback	alternative	both	20	41
D010	Trost W, Trevor C, Fernandez N, Steiner F, Frühholz S.	2024	Live music stimulates the affective brain and emotionally entrains listeners in real time	To investigate the ability of adaptive and dynamic live music to elicit more consistent brain activity in the limbic brain system as well as in the broader neural network for processing musical emotions, we implemented a closed-loop music performance setup for a human neuroimaging environment. Previous setups that connected listeners' brain responses with online music generation algorithms demonstrated general emotional and cognitive effects in listeners	This setup had four main features: 1) piano players were asked to modulate their live music performance on 12 pleasant and unpleasant musical pieces specifically composed for this experiment in order to increase and maximize amygdala activity in listeners in real time; 2) piano music was chosen because piano is a popular and familiar solo instrument, and pianists can play a melody simultaneously with harmonic accompaniment, both important for conveying and inducing emotions; 3) we chose the left amygdala as a target region for neurofeedback setup, as it has been shown to more reliably respond to emotional music than the right amygdala (1, 6); and 4) we chose to compare neural activity during the live music performance with pre-recordings of the same musical pieces by the same pianists as the optimal baseline condition that allows to control for critical features of our experimental design.	Live music also stimulated a dense functional neural network with the amygdala as a central node influencing other brain systems.	we introduce a setup for studying emotional responses to live music in a closed-loop neurofeedback setup.	fMRI	Specifically, during both conditions (live, recorded), we presented the same musical pieces played by the same pianists, with the major difference being the feedback loop (live) compared with the no-feedback condition (recorded). We predicted finding significantly higher neural activity in the limbic target region and the broader neural network for music emotion processing with our setup, compared with a pre-recorded music setup for human neuroimaging, given its adaptive and dynamic nature.	Live pleasant and unpleasant piano music performed in response to amygdala neurofeedback from listeners was acoustically very different from comparable recorded music and elicited significantly higher and more consistent amygdala activity.		27	Original	Live piano music	three distinct performance modulation types, namely, articulation (how a note or musical event is played, also referred to as the expressiveness of the piece), density of note (how many musical events played, also referred as the complexity of the piece) and dynamic (refers to the energy or volume of a sound/note).	1	n/a	left amygdala	https://doi.org/10.1073/pnas.2316306121	feedback	none	new	1	27
